# Supplementary material for: GPR109A mediates the effects of hippuric acid on regulating osteoclastogenesis and bone resorption in mice
Source: Commun Biol. 2021 Jan 8;4:53. doi: 10.1038/s42003-020-01564-2 (PMC7794563; doi:10.1038/s42003-020-01564-2)
Supplement: Supplementary file 2 — Supplementary Information [file 42003_2020_1564_MOESM2_ESM.pdf]

Supplementary Figures

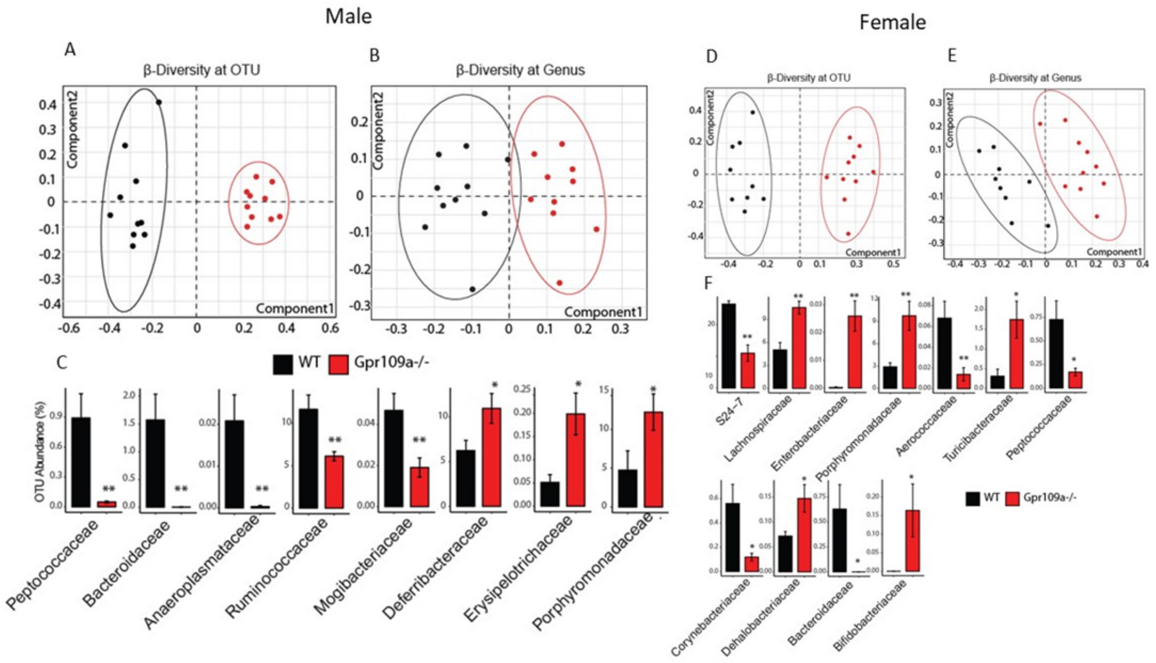

Supplemental Fig. 1. Changes of gut microbiome profiles in GPR109A$^{-/-}$ mice. DNA was isolated from cecal contents. V4 region of bacterial DNA we sequenced using illumina platform. The β-diversity of microbial ecology upon ten weeks old wild type and GPR109A$^{-/-}$ mice. Non-metric multi-dimensional scaling (NMDS) analysis of the operational taxonomical unit (OTU) abundance matrix of the β-diversity of gut microbial communities shows differences at both the (A) phylum and (B) genus level between wild type and GPR109A$^{-/-}$ mice (n=6). (C) The OTU abundance of Peptococcaceae, Bacteroidaceae, Anaeroplasmatacceae, Ruminococcaceae and Mogibacteriaceae was decreased, and Deferribacteraceae, Erysipelotrichaceae and porphyomonadaceae was increased in GPR109A$^{-/-}$ mice compared with their controls. In females, the β-diversity of gut microbial communities shows differences at both the (D) phylum and (E) genus level between wild type and GPR109A$^{-/-}$ mice similar to we found in male mice (n=6). (F) The OTU abundance of S24-7, Aeroccocaceae, Peptococcaceae, Corynebacteriaceae and Bacteroidaceae was significantly decreased, but, Lachnospiraceae, Enterobacteriaceae, Porphyromonadaceae, Turicibacteraceae, Dehalobacteriaceae and Bifidobacteriaceae was significantly increased in GPR109A$^{-/-}$ mice compared with their wild type controls.* $p<0.05$.

Supplementary Fig. 2. Blots shown in Fig.6 with size markers and uncropped blot images.

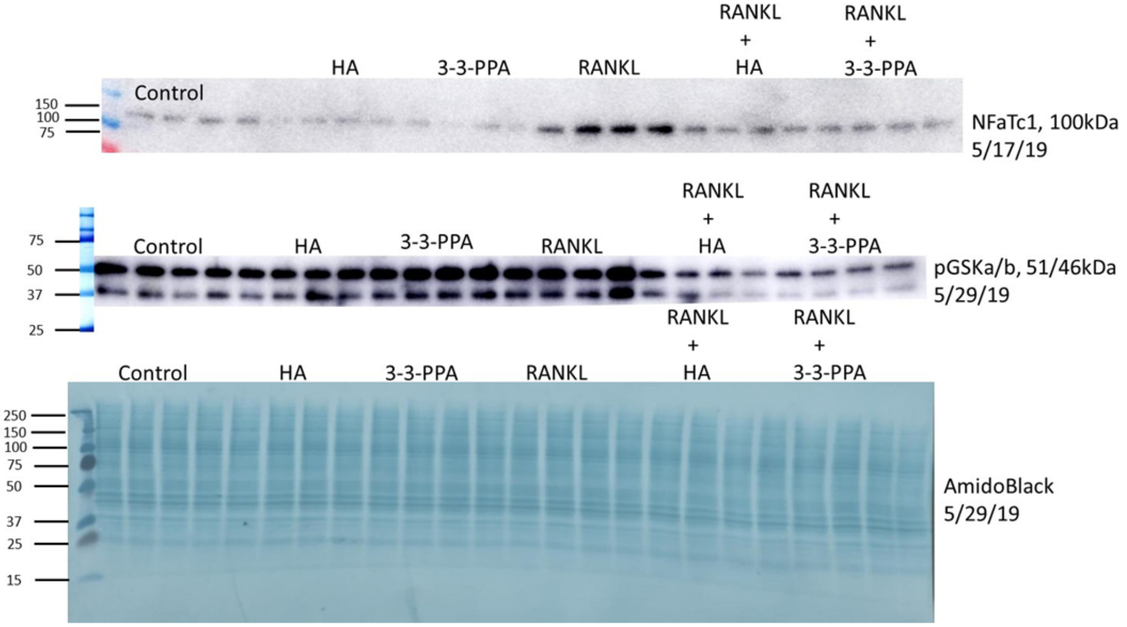

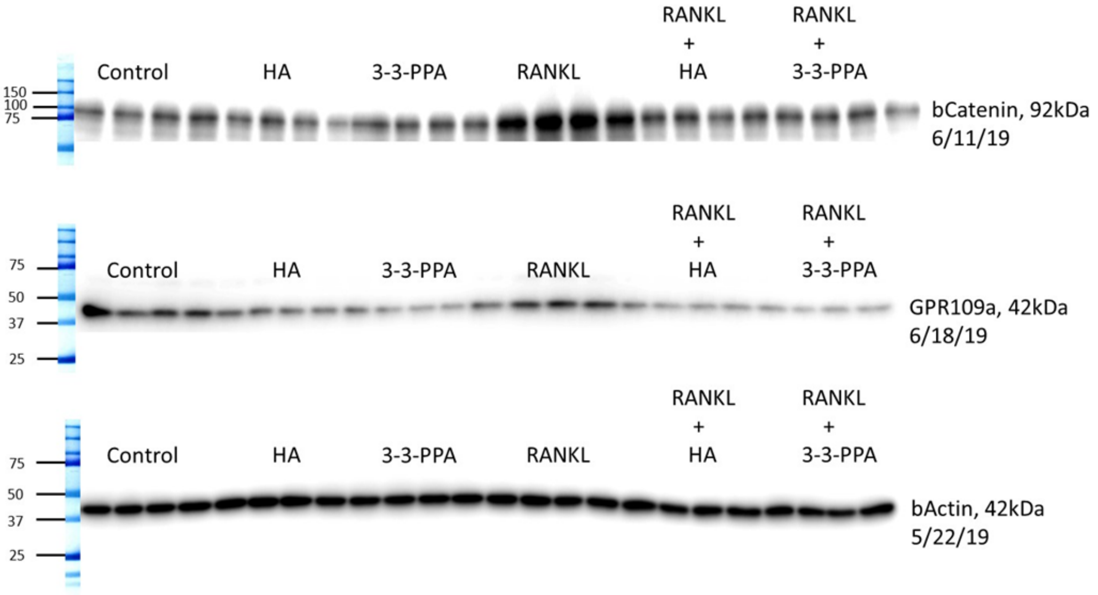

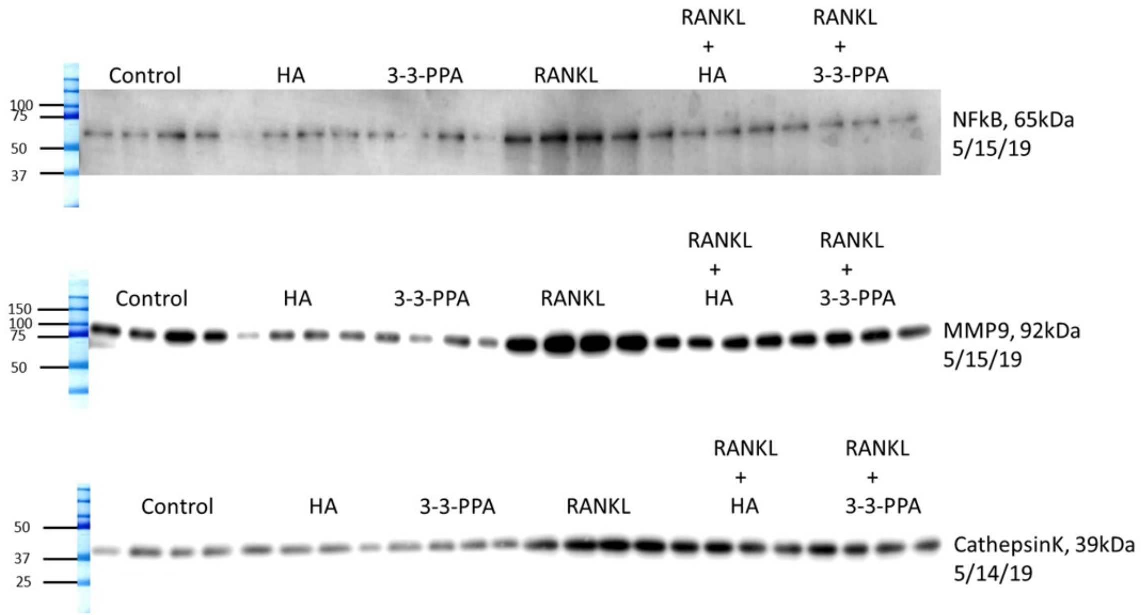
Control
HA
3-3-PPA
RANKL
RANKL + HA
RANKL + 3-3-PPA
100
75
50
37
NFkB, 65kDa
5/15/19
150
100
75
50
MMP9, 92kDa
5/15/19
50
37
25
CathepsinK, 39kDa
5/14/19

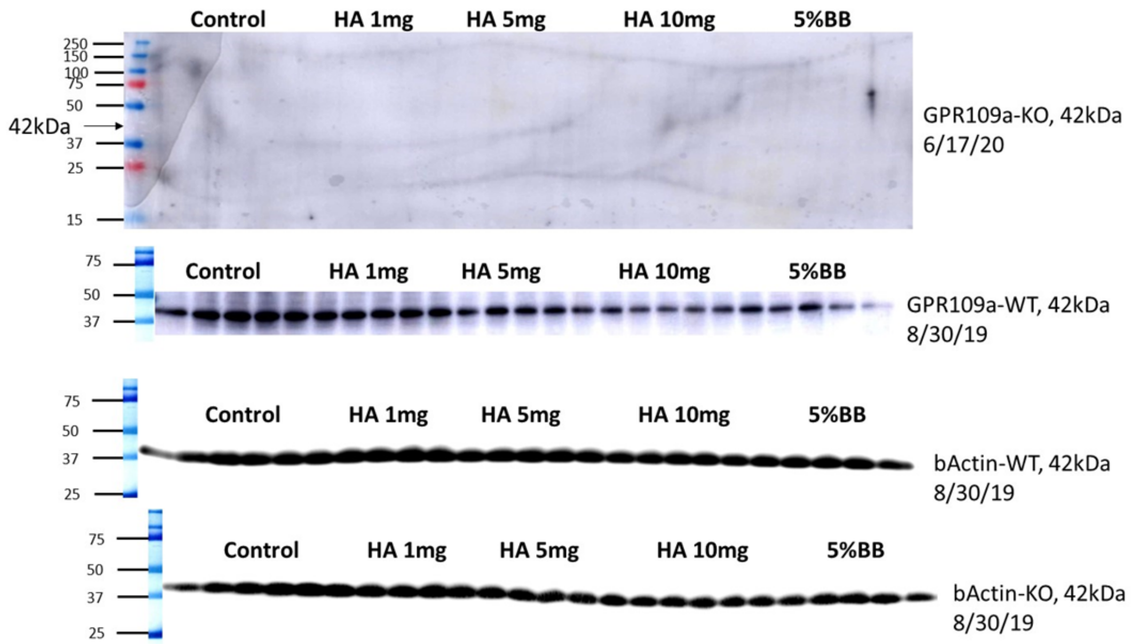
Control
HA 1mg
HA 5mg
HA 10mg
5%BB
250
150
100
75
50
42kDa
37
25
15
GPR109a-KO, 42kDa
6/17/20
75
50
37
GPR109a-WT, 42kDa
8/30/19
75
50
37
25
bActin-WT, 42kDa
8/30/19
75
50
37
25
bActin-KO, 42kDa
8/30/19

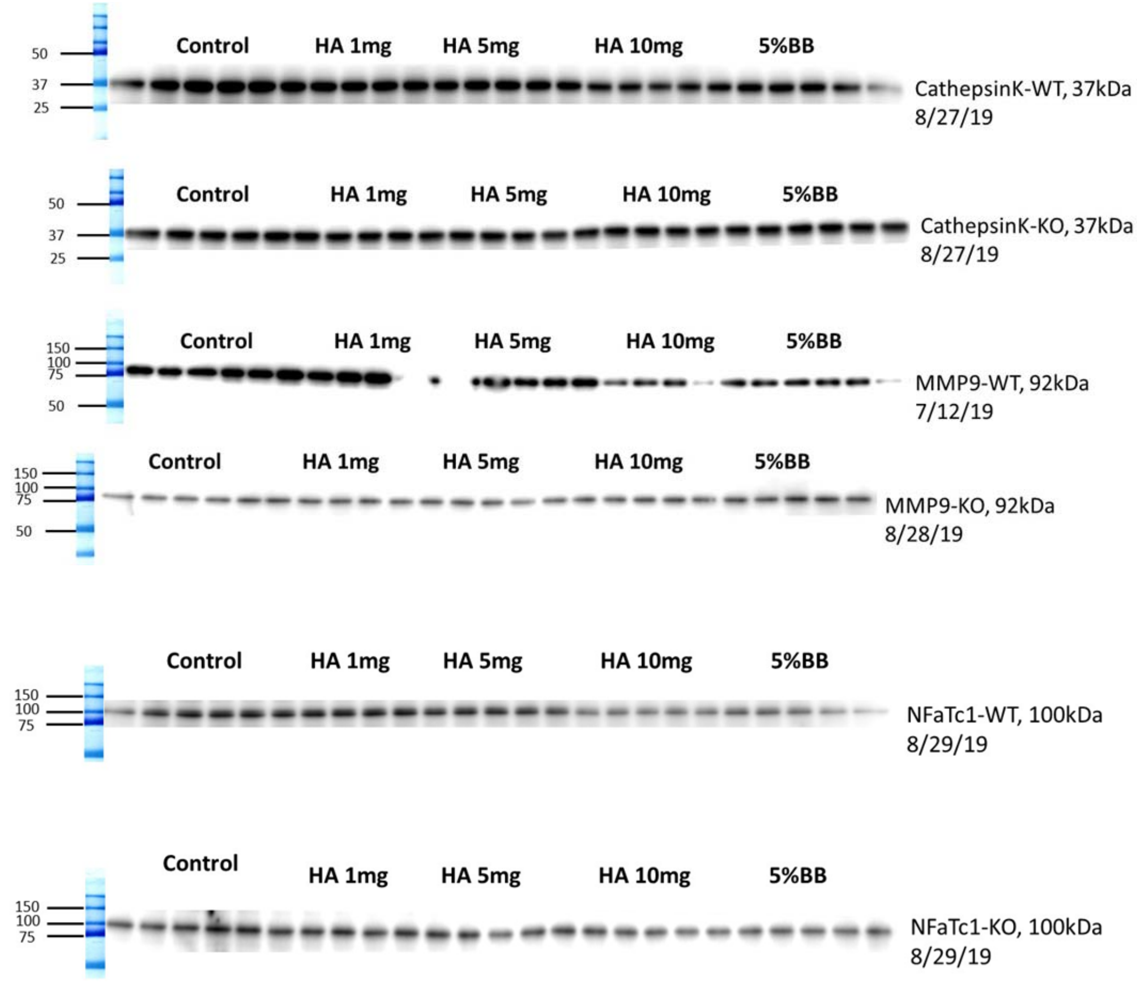

50
37
25
Control
HA 1mg
HA 5mg
HA 10mg
5%BB
CathepsinK-WT, 37kDa
8/27/19
50
37
25
Control
HA 1mg
HA 5mg
HA 10mg
5%BB
CathepsinK-KO, 37kDa
8/27/19
150
100
75
50
Control
HA 1mg
HA 5mg
HA 10mg
5%BB
MMP9-WT, 92kDa
7/12/19
150
100
75
50
Control
HA 1mg
HA 5mg
HA 10mg
5%BB
MMP9-KO, 92kDa
8/28/19
150
100
75
Control
HA 1mg
HA 5mg
HA 10mg
5%BB
NFaTc1-WT, 100kDa
8/29/19
150
100
75
Control
HA 1mg
HA 5mg
HA 10mg
5%BB
NFaTc1-KO, 100kDa
8/29/19

Supplementary Table 1. Chemical reagents, antibodies, proteins, cell lines, mice and assay kits used for this investigation

**Chemical Reagents**	**Source**	**Catalogue #**
Amido Black Staining Solution	Sigma (MO, USA)	A8181
MEM α, nucleosides, no phenol red	ThermoFisher (MA, USA)	41061029
Fetal Bovine Serum (FBS), United States	ThermoFisher (MA, USA)	26140079
Penicillin-Streptomycin (5,000 U/mL)	ThermoFisher (MA, USA)	15070063
Recombinant Mouse TRANCE/TNFSF11/RANK L	R&D Systems (MN, USA)	462-TEC
Acid Phosphatase, Leukocyte Kit	Sigma (MO, USA)	386
Osteo Assay Surface Microplate	Corning (AZ, USA)	CLS3989
Hippuric Acid	Alpha Aesar (MA, USA)	A12690
3-(3-hydroxyphenyl)propionic acid	Alpha Aesar (MA, USA)	L01279
Formalin, Buffered PBS, 10%	FisherScientific (MA, USA)	SF100-4
Glutaraldehyde 2.5% in Phosphate Buffer	FisherScientific (MA, USA)	50-366-996
Von Kossa Stain Kit	Abcam (MA, USA)	ab150687
TRI Reagent™ Solution	Invitrogen (CA, USA)	AM9738
DNase Max Kit	QIAGEN (MD, USA)	15200-50
iScript™ cDNA Synthesis Kit	Bio-Rad (CA, USA)	1708890
Fast SYBR™ Green Master Mix	ThermoFisher (MA, USA)	4385612
RIPA Lysis and Extraction Buffer	ThermoFisher (MA, USA)	89901
SuperSignal™ West Pico PLUS Chemiluminescent Substrate	ThermoFisher (MA, USA)	34577
DNeasy PowerSoil Pro Kit	QIAGEN (MD, USA)	47014
**Antibodies**	**Source**	**Catalogue #**
β-Catenin	BD Transduction Lab	610154
CathepsinK	Abcam (MA, USA)	ab187647
p-GSK3 α/β	Cell Signaling (MA, USA)	9331
GPR109a	US Biological (MA, USA)	223312
MMP9	Millipore (MA, USA)	AB19016
NFaTc1	Sigma (MO, USA)	SAB2101576
NFkB	Sigma (MO, USA)	05-1469
β-Actin	Sigma (MO, USA)	A1978
**Assay Kits**	**Source**	**Catalogue #**
CTX 1	MyBioSource (CA, USA)	MBS722404
P1NP	MyBioSource (CA, USA)	MBS2500076
**Mice**	**Source**	**Catalogue #**
GPR109A$^{-/-}$	Hospital Hygiene Heinrich-Heine-University Düsseldorf, Germany.	

C57BL/6J	The Jackson Laboratory, USA	
**Animal Diet**	**Source**	**Catalogue #**
Blueberry Diet	VDF/FutureCeuticals, Momence, IL	
AIN-93G diet	Harlan Industries, Indianapolis, IN	

Supplementary Table 2. Sequence of primer.

Gene	Sequence (5' – 3')	
	Forward	Reverse
mGPR109a	CGC TGC CTT CGA AAG AAA AC	GCC CCT GGA ATA CTT CTG GTT
mGAPDH	GTA TGA CTC CAC TCA CGG CAA A	GGT CTC GCT CCT GGA AGA TG
mTNFa	GAC GTG GAA CTG GCA GAA GAG	GCC ACA AGC AGG AAT GAG AAG
mTRAP	TGG TCC AGG AGC TTA ACT GC	GTC AGG AGT GGG AGC CAT ATG
mCathepsinK	GTG GGT GTT CAA GTT TCT GC	GGT GAG TCT TCT TCC ATA GC
mDNMT3a	TGCCAATAACCATGACCAGGA	CCCTGTAGCAATCCCATCAAAG
mEZH2	TCC TGG ATG TCG GTG CAA A	CAG CTC CAC ACG TGA GAC AGA

Supplementary Methods

Material and product information is in Supplementary Table 1, and primers used in the study are listed in Supplementary Table 2.

Animals and treatments with 5% BB diet and HA supplementation

GPR109A$^{-/-}$ mice were from Dr. Muthusamy Thangaraju (Department of Biochemistry and Molecular Biology, Georgia Regents University, US) (Elangovan et al., 2014), and this systemic GPR109A$^{-/-}$ mouse model was originally made by Dr. Klaus Pfeffer's research group at the Institute of Medical Microbiology and Hospital Hygiene Heinrich-Heine-University Düsseldorf, Germany (Tunaru et al., 2003) We have inbred GPR109A gene knockouts and C57BL/6J wild type mice to generate male and female GPR109A gene deletion and wild type mice for the current studies. Experiments involved 4-week-old and 6-month-old littermates of male and female GPR109A$^{-/-}$ and their corresponding wild type male and female mice (6 per group) for bone phenotyping studies. Thirty male GPR109$^{-/-}$ mice and thirty male wild type mice were generated for blueberry (BB) diet and HA supplemental diet feeding studies (6 per treatment group). Mice were weighed, randomized by their weights, and housed 6 per cage in mouse small shoe box cages. Control mice received AIN-93G diet formulated with casein as the sole protein source throughout the experiment. The other eight groups of mice received either 5% BB diet or HA (from Alfa Aesar, USA cas#621-54-5) supplemented 1 mg/kg/day (8.4 mg/kg in diet), 5 mg/kg/day (42 mg/kg in diet) and 10 mg/kg/day (84 mg/kg in diet) daily for 40 days, designated as 5% BB, HA 1, 5 and 10 mg groups, respectively. These doses were based on serum HA concentrations of rats fed a BB-containing diet and *in vitro* in cell cultures (Zhao et al., 2020; Chen et al., 2019) and previous *in vivo* subcutaneous HA injection experiments (Chen et al., 2014). All BB diet and HA treatment groups were pair-fed to control, and their food and calorie intakes were matched to control group. Mice were housed in an Association for Assessment and Accreditation of Laboratory Animal Care-approved animal facility in the Arkansas Children's Nutrition Center Animal Studies Core at the Arkansas Children's Research Institute, with constant humidity and lights on from 06:00-18:00 hr at 22°C. All animal procedures were approved by the Institutional Animal Care and Use Committee at University of Arkansas for Medical Sciences (AUP#3595 UAMS, Little Rock, AR). At the end of the studies, mice were anesthetized

by injection with 100 mg Nembutal/kg body weight (Avent Laboratories). Blood was collected via cardiac puncture, which was followed by decapitation, femur, tibia and vertebrae bones were collected and stored in -80 ºC.

Bone analysis using micro-CT and peripheral quantitative computerized tomography (pQCT) and biomechanical testing three-point bending

Micro-computed tomography measurements of the trabecular and cortical compartments from the left tibial and L5 spine bone were evaluated using SkyScan μCT scanner (recently upgraded SkyScan 1272, Bruker.com) at 8 μm pixel size with X-ray source power of 60 kV and 166 μA and integration time of 950 ms. The trabecular compartment included a 0.9 mm region extending distally 0.03 mm from the physis. The grayscale images were processed by using a filter (=Al, σ=0.5, mm) to remove noise, and a fixed threshold of 125 was used to extract the mineralized bone from the soft tissue and marrow phase. Cancellous bone was separated from the cortical regions by semi-automatically drawn contours. A total of 100 slices starting from about 0.1 mm distal to growth plate, constituting 0.80 mm length, were evaluated for trabecular bone structure, bone volume fraction (BV/TV, %), trabecular thickness (Tb.Th, mm), trabecular separation (Tb.Sp, mm), trabecular number (Tb.N, 1/mm), Degree of anisotropy (DA) were calculated based on description by Bouxsein et al.(Bouxsein et al., 2010; Chen et al., 2013), and by using software provided by SkyScan, Bruker. For cortical bone, the cortical compartment was a 0.6 mm region extending distally starting 5 mm proximal to the tibia-fibula junction. Total cross-sectional area (CSA, mm^2), medullary area (MA, mm^2) and cortical thickness (Ct.Th, mm) were assessed.

Peripheral quantitative computerized tomography (pQCT) was performed on formalin fixed left tibia for bone mass bone mineral density (BMD) measurement using a method established in our laboratory (Chen et al., 2017; Zhang et al., 2011). A STRATEC XCT 960 M unit (XCT Research SA, Norland Medical Systems, Fort Atkins, WI) specifically configured for small bone specimens was utilized. Software version 5.4 was used with thresholds of 570 mg/cm3 to distinguish cortical bone and 214 mg/cm3 to distinguish trabecular from cortical and sub-cortical bone. Tibial BMD and bone mineral content (BMC) were calculated. The position for pQCT scanning was defined at a distance from proximal tibia 1 mm below growth plate corresponding to 7% of the total

length of the tibia. Distance between each scanning was 0.5 mm, total of 5 scans (five slices) were carried out. Data were expressed as the mean of three contiguous slices with the greatest trabecular bone density.

Three-point bending test was performed on femurs at room temperature using a miniature bending apparatus with the posterior femoral surface lying on lower supports (7 mm apart) and the left support immediately proximal to the distal condyles. Load was applied to the anterior femoral surface by an actuator midway between the two supports moving at a constant rate of 3 mm/min to produce a physiological *in vivo* strain rate of 1% for the average murine femur. Maximum load (N) and displacement (mm) were recorded. The external measurements (length, width and thickness) of the femora were recorded with a digital caliper. We measured the moment of inertia in the midshaft of femur using µCT (model µCT40, Scanco Medical). The mechanical properties were normalized for bone size and ultimate strength and stress (N/mm2; in megapascals and MPa) was calculated.

Bone histology

Mouse right tibia samples were embedded, cut and TRAPase stained by Histology Special Procedures at the ACNC Histology Core. TRAPase staining kit was utilized according to the manufacturer's protocols (Sigma-Aldrich, Acid phosphatase leukocyte, procedure No. 386). TRAPase positive pink-stained cells were visualized with a digitizing morphometry system, which consists of an epifluorescent microscope (model BH-2, Olympus), a color video camera, and a digitizing pad (Numonics 2206) coupled to a computer (Sony) (OsteoMetrics, Inc.).

Measurements of bone turnover markers in bone marrow plasma and in serum

Bone marrow plasma were prepared at the time of tissue harvest. Bone marrow was flushed out from femur using 300 µl of PBS, vortexed and spun (1700 g) for collecting supernatant as bone marrow plasma. The bone resorption marker C-terminal telopeptides of type I collagen (CTX-1) was measured by ELISA kit was purchased from Mybiosource.com (Catalog No: MBS722404). The bone marrow plasma and serum P1NP (Procollagen 1 N-terminal Propeptide) levels were measured by direct immunoassay P1NP assay Kit. The P1NP level measurement ELISA kit was purchased from Mybiosource.com (Catalog No: MBS2500076) and measurement procedure

followed the manufacturer's recommendations. According to the protocol, pre-coated with total-P1NP antibody, total-P1NP present in the sample is added and binds to antibodies coated on the wells, and then biotinylated total-P1NP antibody is added and binds to total-P1NP in the sample. Then, streptavidin-HRP is added and binds to the biotinylated total-P1NP antibody. After incubation unbound streptavidin-HRP is washed away during a washing step, substrate solution is then added and color develops in proportion to the amount of total-P1NP. The reaction is terminated by addition of acidic stop solution and absorbance is measured at 450 nm.

Cell cultures

Non-adherent bone marrow cells were cultured in 96-well plates (2×10^4 cells/well) in the presence or absence of 50 ng/ml of RANKL, in α-MEM supplemented with 10% fetal bovine serum (FBS) (ThermoFisher, MA, USA), penicillin (100 Units/ml), streptomycin (100 μg/ml), and glutamine (4 mM). These cell cultures were previously described in our laboratory[47]. After 5 days for bone marrow cell cultures, the cells were fixed with 4% paraformaldehyde and stained for TRAPase activity using a TRAPase staining kit according to the manufacturer's protocols (Sigma-Aldrich, Acid phosphatase leukocyte, procedure No. 386). TRAP-positive cells containing >3 nuclei in each well were counted as osteoclasts under an epifluorescent microscope (model BH-2, Olympus, Imaging America Inc.; Center Valley, PA). For osteoclast resorption activity assay, non-adherent bone marrow cells were seeded in triplicate per animal in 6-well collagen-coated plates (BD Biosciences) at a density of 1×10^5 cells/well, and cells were treated with 50 ng/ml RANKL for 2 to 3 days. On day three when osteoclasts started to differentiate to mature cells, we dissociated the cells and cultured the same number of osteoclastic cells onto hydroxyapatite-coated plates (CLS3989, Corning). Cells in the culture plates were fixed using 2.5% glutaraldehyde with or without Von Kossa staining. The areas of hydroxyapatiter esorption were observed by light microscopy and analyzed using Image J software.

RNA isolation, real-time reverse transcription-polymerase chain reaction

Bone marrow cell RNA isolation was performed using TRI Reagent (ThermoFisher, MA, USA) according to the manufacturer's recommendations followed by DNase digestion and column cleanup using QIAGEN mini columns (Zhang et al., 2014). Reverse

transcription was carried out using an iScript cDNA synthesis kit from Bio-Rad (Hercules, CA). All primers for real-time PCR analysis used in this report were designed using Primer Express software 2.0.0 (Applied Biosystems) and listed in Supplementary Table 2.

Western blotting

Total protein extracts from L3 and non-adherent bone marrow cells after treatment with HA or 3-3-PPA were prepared using radioactive immunoprecipitation assay (RIPA) buffer (Solarbio). Western blots were performed using standard protocols[20]. The protein lysates were quantified and separated by sodium dodecyl sulfate polyacrylamide gel electrophoresis and transferred to polyvinylidene fluoride membranes (Millipore). This was followed by immunoblotting with primary antibodies NFATc1 (SAB2101576, Sigma-Aldrich, St. Louis, MO, USA), MMP9 (AB19016, Millipore, MA, USA), Cathepsin K (ab187647,Abcam, MA, USA), GPR109A (#223312, US Biological, MA, USA), β-catenin (BD Transduction Lab, #610154), pGSK3α/β (Cell signaling, #9336), and β-Actin (A1978, Sigma-Aldrich, St. Louis, MO, USA); 1:1000 dilution and then by the corresponding horseradish peroxidase conjugated secondary antibodies. Blots were developed using chemiluminescence (PIERCE Biotechnology) according to the manufacturer's recommendations. Bands of interest were visualized and imaged under chemiluminescent detection using a Amersham Imager 600 System, GE. Quantitation of the intensity of the bands in the autoradiograms was performed using a VersaDocTM imaging system (Bio-Rad).

Microbial community profiling using 16S rRNA amplicon sequencing and bioinformatics analysis

Using previously published methods (Wankhade et al., 2019), genomic DNA was extracted from the cecal contents using the MO BIO PowerSoil DNA Isolation kit (Qiagen, Gaithersburg, MD, USA). The cecal contents (20–25 mg) were added directly into 96-well plates with beads and recommended buffers in the wells. The sealed plates were shaken horizontally at 20 rpm for 20 min using the MO BIO shaker. The remaining steps were performed as directed by the manufacturer. The extracted DNA was quantitated spectrophotometrically and stored at -20℃. Fifty nanograms of genomic DNA were utilized for the amplification of the V4 variable region of the 16S rRNA gene

using 515F/806R primers. Forward and reverse primers were dual-indexed, as described by Kozich et al., to accommodate the multiplexing of up to 384 samples per run (Kozich et al., 2013). Paired-end sequencing (2 x 250 bp) of pooled amplicons was carried out on an Illumina MiSeq with ~30% of PhiX DNA. The processing and quality-filtering of reads were performed by using scripts in QIIME (v1.9.1) (Caporaso et al., 2010) and other in-house scripts. Paired reads were stitched with PEAR, an over-lapping paired-end reads merger algorithm, which evaluated all possible paired-end read overlaps, minimizing false-positive hits (Zhang et al., 2014). With details we published recently (Wankhade et al., 2017), PICRUSt, a bioinformatics software package designed to predict metagenome functional content from marker gene surveys (e.g., 16S rRNA) and full genomes, was used to identify differences in predictive metagenome function (Langille et al., 2013).

Statistical analyses

Statistical power was computed based on a two-factor ANOVA with 6 mice per group. In our preliminary analysis of the effects of 5% blueberry diet supplementation, or HA injection using a sample size (8 rats/group, 5 mice/group) smaller than in the proposed study, the ES(f) from comparing tibial cortical BMD between blueberry diet-fed rats to those on normal chow was 0.89, whereas an ES(f) =1.34 was observed when comparing tibial trabecular bone mineral density (BMD) between the two groups. Hence, with a sample size of 6/group, we should have sufficient power to detect meaningful differences. Numerical variables were expressed as means ± SEM (Standard Error of Mean). Comparisons between groups were performed with the nonparametric Kruskal-Wallis test followed by a Dunnett’s test comparing each dose to the control group. The nonparametric Wilcoxon rank-sum test was used to compare controls to individual treatment. Cell culture experiments were conducted at least three independent times, and representative images are displayed. Dose-response was assessed using Cruick’s non-parametric test for trend. The critical p-value for statistical significance was $p = 0.05$.
